# Radiology and the medical student: do increased hours of teaching translate to more radiologists?

**DOI:** 10.1259/bjro.20210074

**Published:** 2021-12-08

**Authors:** Cindy Chew, Patrick J O'Dwyer, David Young

**Affiliations:** 1 School of Medicine, Dentistry and Medicine, University of Glasgow, Glasgow, United Kingdom; 2 Department of Radiology, University Hospital Hairmyres, Glasgow, United Kingdom; 3 Department of Mathematics and Statistics, Strathclyde University, Glasgow, United Kingdom

## Abstract

**Objectives:**

The UK has a shortage of Radiologists to meet the increasing demand for radiologic examinations. To encourage more medical students to consider Radiology as a career, increased exposure at undergraduate level has been advocated. The aim of this study was to evaluate if formal Radiology teaching hours at medical school had any association with the number of qualified Radiologists joining the General Medical Council Specialist Register.

**Methods:**

Total number of doctors joining the GMC Specialist Register as Clinical Radiologists, and those with a primary medical qualifications awarded in Scotland, was obtained from the GMC (2010–2020). Graduate numbers from all four Scottish Medical Schools (2000–2011) were also obtained. Hours of Radiology teaching for medical schools in Scotland were obtained from validated AToMS study.

**Results:**

Two hundred and twenty three (6.6%) of 3347 Radiologists added to the GMC Specialist Register between 2010 and 2020 received their primary medical qualification (PMQ) from Scottish Universities. The number of Radiologists from Scottish Universities joining the GMC specialist register was 2.6% of the total number of Scottish Medical Graduates. There was no association between the number of hours (Range 1–30) Radiology was taught to medical students and the number that joined the specialist register as Radiologists (*p* = 0.54 chi square trend).

**Conclusion:**

Increased exposure to Radiology teaching does not influence medical students’ decision to take up Radiology as a career. While continued Radiology exposure remains important, other strategies are required in both the short and long term to ensure radiology services are maintained without detriment to patients.

**Advances in knowledge:**

Increased hours of Radiology teaching in medical school was not associated with increased radiologists joining the profession.

## Introduction

There is ever increasing demand for radiologic examinations juxtaposed against a shortage of radiologists. At February 2021, 138,385 patients in England were waiting over six weeks for a CT, MRI or ultrasound scan – 12 times higher than in February 2020.^1^ Similarly in Wales, 20,548 patients were waiting over eight weeks – 33 times more than 2020.^2^ In Scotland, the number of patients waiting over 6 weeks for radiological examinations are 6491 and 21,338 in the same periods.^3^ The Royal College of Radiologists estimates that if current trends continue, there will be 44% shortfall in UK’s radiologists by 2025 or 3,613 consultants in real terms demand.^4^ This finding is reflected worldwide.^5^ A large increase in newly qualified doctors entering Radiology training will be needed to meet this shortfall. One way to encourage this may be to increase medical student exposure to radiology in both the preclinical and clinical years with formal teaching hours dedicated to the subject – by radiologists.

More medical school teaching in some subjects have been shown to result in an increase in trainees for some specialties, but not for others.^6^ The impact for Radiology has not been assessed.

The aim of this study is to evaluate if formal dedicated radiology teaching hours at medical school had any association with longitudinal data on qualified Radiologists joining the General Medical Council’s (GMC) specialist medical register at a national level.

## Methods

Information on the total number of doctors joining the GMC Specialist Register as Clinical Radiologists, as well as the subset number of those with a primary medical qualifications awarded in Scotland, was obtained from the GMC through the Freedom of Information Act over a 10 year period (2010–2020). Graduate numbers from all four Scottish Medical Schools (2000–2011) were also obtained from the GMC, as this group of medical graduates would likely join the Specialist Register between 2010 and 2020.

Hours of Radiology and total hours of teaching was obtained from the AToMS study, pertaining to all timetabled teaching in 2015.^7^ These timetables were previously validated against the Higher Education Policy Institute (HEPI) Student Academic Experience Survey as well as the study of teaching of general practice, which collected data from heads of Departments of General Practice in UK medical schools.^7^

Information on FRCR pass rates was obtained from the GMC Specialty Examination Reporting Tool website.^8^

Information on historic application to Radiology was obtained from the Royal College of Radiologists website.^9^

Assuming an average of 9 years (with a minimum of 7 years) between graduating from medical school and entering the specialist register, we also set to determine if medical graduate numbers remained stable between 2001 and 2011 to correct for any impact this may have on numbers joining the specialist register during the study period. To compare the portions becoming Radiologists in Scotland over the time period 2010–2020, a chi-squared goodness to fit test was performed. A two-way ANOVA test was used to investigate the difference in FRCR pass rates between the Scottish Universities from 2014–2019.

## Results

### Radiologists joining GMC specialist register (2010–2020)

Two hundred and twenty three (6.6%) of 3347 Radiologists added to the GMC Specialist Register between 2010 and 2020 received their primary medical qualification (PMQ) from Scottish Universities (Table 1). The number of doctors joining the specialist register from Scottish PMQ Awarding bodies did not change significantly (chi squared trend *p* = 0.543) over the 10 year period (Table 1). The number of medical students graduating from Scottish Medical schools remained stable with no significant change during 2001–2011 – the period in which this group of Radiologists is likely to have graduated. The number of Radiologists from Scottish Universities joining the GMC specialist register was 2.6% of the total number of Scottish Medical graduates (Table 2).

**Table 1.** Radiologists joining the GMC Specialist Register 2010–2020

	**2010** (% of total)	**2011** (% of total)	**2012** (% of total)	**2013** (% of total)	**2014** (% of total)	**2015** (% of total)	**2016** (% of total)	**2017** (% of total)	**2018** (% of total)	**2019** (% of total)	**2020** (% of total)	Total (% of total)
Radiologists added to Specialist Register	292	275	291	292	380	292	299	294	298	307	327	3347
Radiologists with Scottish PMQ	22 (7.5)	21 (7.6)	23 (7.9)	23 (7.9)	12 (3.2)	23 (7.9)	12 (4.0)	22 (7.5)	20 (6.7)	21 (6.8)	24 (7.3)	223 (6.7)

**Table 2.** Total number of Medical Students Graduating from Scottish Medical Schools

Year / Medical School	Aberdeen	Dundee	Edinburgh	Glasgow	Total Scottish Graduates
2001	158	131	195	194	678
2002	159	131	239	269	798
2003	183	161	176	256	776
2004	167	137	218	190	712
2005	185	147	252	246	830
2006	176	144	219	250	789
2007	193	159	227	240	819
2008	200	155	261	245	861
2009	171	158	251	243	823
2010	183	152	238	236	809
2011	186	129	230	270	815
**Total**	**1961**	**1604**	**2506**	**2639**	**8710**

The number of radiologists added to the specialist register in the UK as a whole mirrored that of Scotland, in that there was no change in the number over the same period (Table 1).

### Radiology teaching hours and radiologists joining GMC specialist register

Glasgow is the largest and only Problem-Based Learning School in Scotland. It had lowest total recorded formal teaching hours but the highest hours of radiology teaching (Table 3).

**Table 3.** Number of Teaching hours correlated to number of Radiologists

Medical School	Total number of Radiologist (2010–**2020**)	Total number of Students (2001–**2011**)	Total Radiology Teaching Hours	Total Teaching Hours
Aberdeen	38	1961	9	5069
Dundee	39	1604	19.1	5446
Edinburgh	68	2506	1	4828
Glasgow^*a*^	78	2639	30	3982

a Glasgow was the largest medical school with 35% more students on average than Aberdeen, 65% more than Dundee and 5% more than Edinburgh.

**Figure 1.**
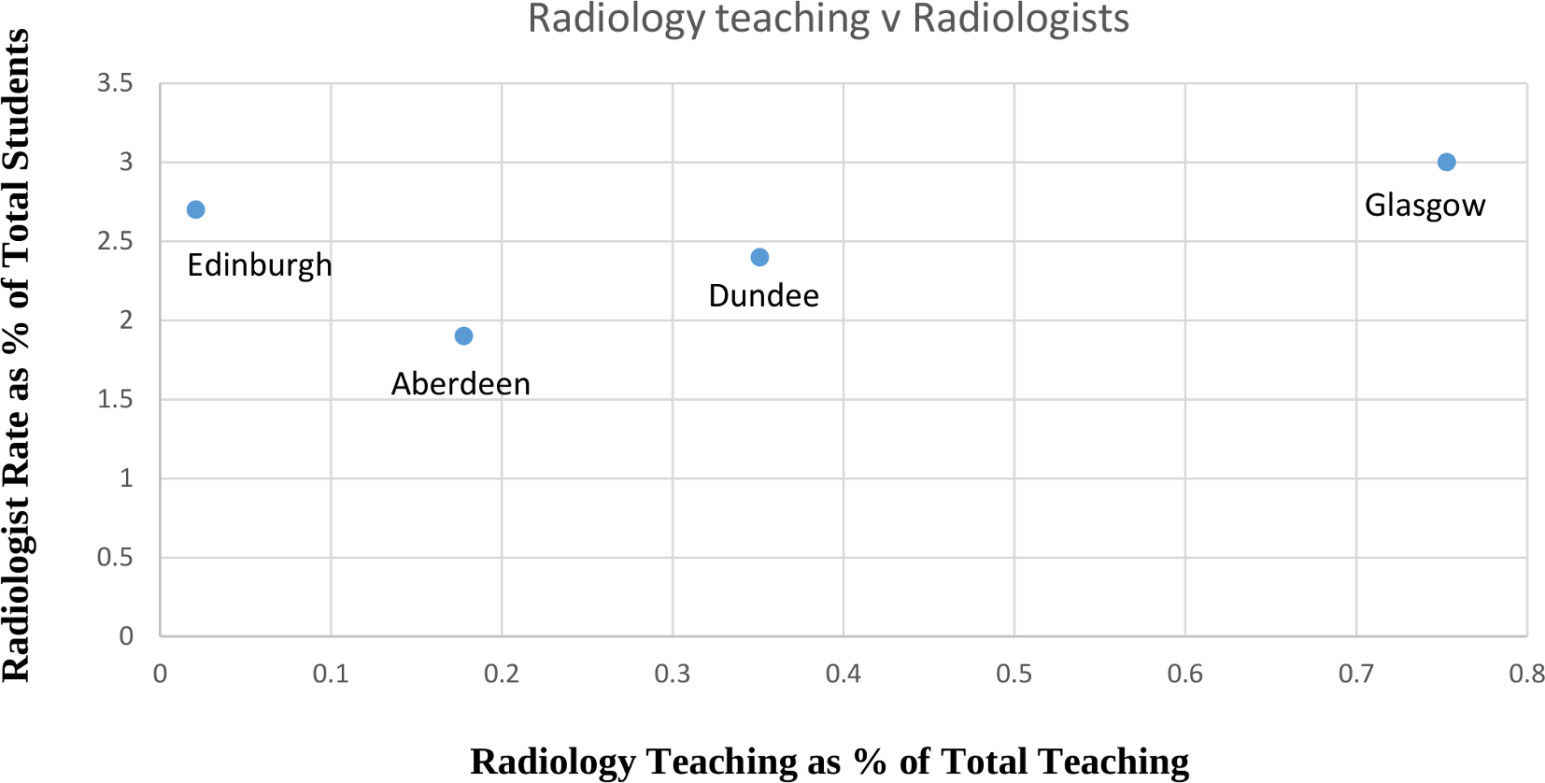
Percentage Radiology teaching plotted against Radiologist Rate for each University

There was no association between the number of formal hours Radiology taught to medical students (range 1–30) and the number that joined the specialist register as Radiologists (Table 3, Figure 1). In addition, there was no association between total hours taught and the numbers of graduates joining the GMC Radiology specialist register.

**Figure 2.**
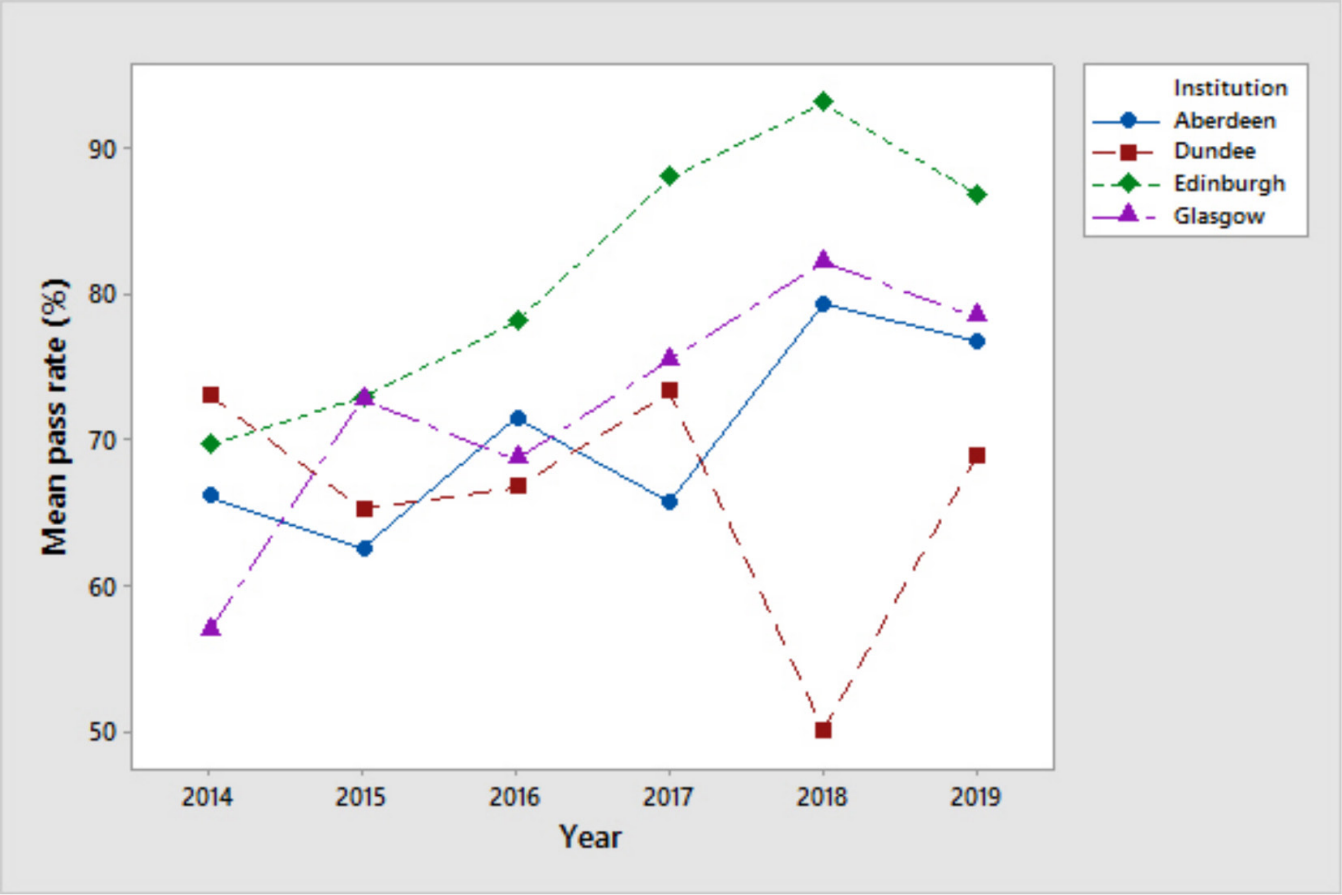
Analysis of Pass rates of each University over time. This was done using two-way ANOVA: There was no significant difference in pass rates over time (p=0.321). There was evidence of a difference between institutions (p=0.034), with Edinburgh pass rates being significantly higher than Dundee (p=0.033)

Edinburgh had the lowest hours of Radiology teaching, but the best pass rates for Radiology examinations (Table 4, Figure 2).

**Table 4.** Analysis of Pass rates of each University over time

Difference of Institution Levels	Difference of Means	SE of Difference	Simultaneous 95% CI	T-Value	Adjusted *P*-Value
Dundee - Aberdeen	−4.10	4.70	(−18.37, 10.17)	−0.87	1.000
Edinburgh - Aberdeen	11.13	4.70	(−3.13, 25.40)	2.37	0.190
Glasgow - Aberdeen	2.05	4.70	(−12.22, 16.32)	0.44	1.000
Edinburgh - Dundee	15.23	4.70	(0.97, 29.50)	3.24	0.033
Glasgow - Dundee	6.15	4.70	(−8.12, 20.42)	1.31	1.000
Glasgow - Edinburgh	−9.08	4.70	(−23.35, 5.18)	−1.93	0.434

Individual confidence level = 99.17%.

Bonferroni Simultaneous Tests for Differences of Means.

## Discussion

This study shows no association between hours of radiology taught in medical schools with the number of radiologists joining the specialist register. Also, despite the well-publicised shortage of radiologists, there had been no increase in those joining the speciality register over the last decade. This is occurring against a background of unrelenting increase in demand for radiology investigations, pre-COVID, in the same period. The 2020 Independent Review of Diagnostic Services for NHS England recommended major expansion of the imaging workforce is required as part of its Recovery and Renewal Plan, with an increase of 2000 Radiologists to meet the demand.^10^

There is apparent face validity to the concept that without any radiology teaching and exposure in medical school, medical students are less likely to consider radiology as a career option and more contact hours could increase interest in Radiology. McManus et al demonstrated in their extensive study that while more GP teaching resulted in more graduates entering GP training, paradoxically, those very graduates performed less well in MRCGP examinations.^6^ Conversely, more teaching in Psychiatry, Surgery and Anaesthetic did not result in more specialist trainees. While Europe reports high levels of Radiology input in their medical student curriculum, only about 20% of US and 6% of Canadian medical schools have Radiology clerkships with a similarly low level of Radiology exposure reported for the United Kingdom.^11,12^ Radiology foot print in Scottish medical schools is also extremely low at 0.3% of the curriculum hours dedicated to radiology.^13^ Against a worldwide shortage of radiologists, radiological societies are working hard to increase the medical school presence of radiology in the hope of improving this shortage. In some parts of Scotland, the estimated workforce shortfall is already over 40%.^4^

Radiology is a vital part of modern medicine – where almost every clinical condition is reliant on a radiological examination for patient management. While we are a valued added specialty, we are also now potentially a bottle neck in patient pathways – heralding patient safety issues.^14^ More needs to be done to recruit radiologists – a report by the Royal College of Radiologists cites the amount NHS Scotland spent on outsourcing radiology quadrupled over 5 years to £4.5 million in 2019–50% of total Scottish Radiologist salaries^15^!

All this is particularly surprising, given Medscape’s 2021 Radiologist Compensation report finding Radiologists are among the highest earners, spend the least time on administration and paperwork, as well as being one of those most likely to choose the same specialty again.^16^

So how can we increase the number of Radiologists in the UK if having more teaching hours in medical school is not apparently effective? Given the scale of the problem, the solution needs to be a political one. One way is to increase the overall total number of medical students and hope the proportion of students choosing Radiology is maintained. The Scottish Government is committed to maintaining a sustainable medical workforce. To that end they have increased medical student places by 22% (190) between 2016 and 2021 as well as enacted policies which will increase the number of Scottish over other UK domiciled medical students during this period to help retain medical school graduates in Scotland for the longer term.^17^

Ultimately, more post-graduate training places need to be made available. The number of people applying to train in Radiology between 2010 and 2021 has doubled with insufficient increase by Government in training places available (Table 5). Radiology could do well to advocate for our specialty and lobby politicians effectively as General Practice has done – as evidenced by the visible investment by government into promoting General Practice in medical schools according to recommendations made by the Gillies report.^18^ This is particularly notable in Scotland where, despite Scottish population making up approximately 8.1% of the total UK population, graduates from Scottish Medical Schools make up just 6.7% of Radiologists joining Specialist register – that is a cumulative annual shortfall of 21% in just the last decade compared to the rest of the UK.^19^

**Table 5.** Number of Applicants per Post in Radiology (2010–2021)

Year	Posts Available	Applicants	Applicants : Post Ratio
2010	185	655	3.54 : 1
2011	183	588	3.21 : 1
2012	167	693	4.14: 1
2013	210	754	3.59: 1
2014	229	794	3.47: 1
2015	244	917	3.76 : 1
2016	249	963	3.87: 1
2017	267	937	3.51: 1
2018	282	970	3.44: 1
2019	278	967	3.48: 1
2020	311	1128	3.62: 1
2021	353	1677	4.75: 1
Mean			3.7 : 1

Source : https://www.rcr.ac.uk/clinical-radiology/careers-and-recruitment/specialty-recruitment/statistical-summary-previous-rounds

Growing Radiologists “from scratch” is often deemed to take too long. A word of caution then of the Scottish Government’s experience. An ambitious international recruitment drive was launched in 2018, as part of its £4 million Scottish Radiology Transformation Program, in an attempt to increase Radiologist numbers quickly. This netted just five recruits, one of whom was already working in Scotland.^20^ Against a worldwide shortage of Radiologists, unfortunately there is no “magic Radiologist-tree” to shake for instant mass hordes of Radiologists. Until government policies, funding and requisite time for training new Radiologists materialise, Health Boards would do well to keep their existing staff Radiologists professionally satisfied not burnt out, to meet the anticipated tsunami of imaging demand on the covid recovery road ahead.

What options are available to the Health Care system in the mean time? Skills mix – advanced practitioners/reporting radiographers – has been the go-to solution for a while. Unfortunately, they are not the panacea, with the Government’s Diagnostic Recovery document highlighteing the need for 4000 additional radiographers to meet the anticipated demand.^9^ Artificial intelligence in its multiple potential forms is not yet ready for mass roll out. The sustainable option is to educate clinicians, and patients, to be judicious with their use of Radiology services to temper demand. This work needs to start right at the Medical Student level. Otherwise, the only – most difficult and contentious – avenue left is to ration Radiology resource.^21,22^ Just as Health Boards and Trust manage running costs by limiting the number of beds in hospitals, so too a cap in the capacity to curb demand may be required to ensure the most urgent and clinically impactful examinations are performed and reported in a timely fashion. Currently only 1% of NHS Trusts and Health Boards are able to report all scans within Radiologists’ contracted hours.^15^ With increasing reliance on outsourcing firms and a rapidly diminishing pool of Radiologists, cost of outsourcing will undoubtedly escalate in response to free market forces.

There are a few limitations to this study. The data relating to hours of teaching in radiology was only from a single year (2015). Information regarding curriculum is notoriously difficult to acquire. This data, acquired through Freedom of Information, was the most comprehensive to date and was validated prior to analysis. Further, medical school curricula are relatively stable entities over time and this data is representative of the current curricula. It is possible the analysis under estimated the true hours of radiology teaching secondary to the way data was collated, particularly during clinical placements. Nonetheless, *ad hoc* radiology teaching was not uniformly available to all medical students in a standardised way and would be difficult to include in our evaluation. Finally, this study only included data from four Scottish Medical Schools, including just 1 PBL school. While this is a relatively small number, the findings are similar to that observed for Psychiatry, Anaesthesia and Surgery for the UK as a whole.^6^

## Conclusion

There has been very little change in the number of radiologists produced from Scottish Medical Schools, and indeed across the United Kingdom, over the last decade. More medical school Radiology teaching did not result in more Radiologists. In the face of massive shortage of radiology workforce, urgent work is needed to address this issue as a matter of patient safety.
